# Exosomal microRNAs as biomarkers of multimorbidity risk in lean and obese women with polycystic ovary syndrome

**DOI:** 10.3389/fmolb.2026.1868855

**Published:** 2026-10-01

**Authors:** Debarati Halder, P. V. Varshini, Tridip Mitra, A. H. Sruthi Anil Kumar, Piyush Agrawal, Mrinmoy Das, A. Senthil Kumar, R. Sajeetha Kumari, S. N. Meenakshi Sundari, Rajiv Janardhanan

**Affiliations:** 1 Division of Medical Research, Faculty of Medicine and Health Sciences, SRM Institute of Science and Technology, Kattankulathur, Tamil Nadu, India; 2 Department of Radio Diagnosis, Faculty of Medicine and Health Sciences, SRM Institute of Science and Technology, Kattankulathur, Tamil Nadu, India; 3 Department of Obstetrics and Gynecology, Faculty of Medicine and Health Sciences, SRM Institute of Science and Technology, Kattankulathur, Tamil Nadu, India; 4 Department of General Medicine, Faculty of Medicine and Health Sciences, SRM Institute of Science and Technology, Kattankulathur, Tamil Nadu, India

**Keywords:** biomarker, early detection, exosomal microRNA, multimorbidity, polycystic ovary syndrome

## Abstract

Polycystic ovarian syndrome (PCOS) is a multifactorial endocrine disorder that is characterised by reproductive, metabolic and cardiometabolic abnormalities. Besides typical hormonal abnormalities, inter-organ communication has a crucial role in the development of PCOS-associated multimorbidity. PCOS causes early changes in the insulin signalling pathway, inflammatory pathways, androgen synthesis genes, and ovarian theca cell activity even before the appearance of regular symptoms such as irregular periods, polycystic ovaries, weight gain, and hyperandrogenism. Exosomes are tiny extracellular vesicles discharged into the circulation. It transports molecular cargoes such as microRNAs (miRNAs), proteins that influence gene expression and reflect the underlying pathophysiological condition of target tissues. Exosomes facilitate early disease detection by carrying stable, selectively packaged gene-regulatory molecules that reflect early cellular and molecular alterations before phenotypic manifestations occur. Although exosomal miRNAs (ExomiR) are stable, non-invasive, and easily detectable in body fluids, they serve as a potent biomarkers. These circulating ExomiR have contributed to multiple organ dysfunctions, including the ovary, adipose tissue, liver, cardiovascular system, and brain, thereby raising the risk of comorbid conditions like endometrial disorder, insulin resistance, type 2 diabetes, non-alcoholic fatty liver disease, and cardiovascular disease. In obese PCOS, circulating ExomiR influences pathways related to steroidogenesis, insulin signalling, inflammation, oxidative stress, and lipolysis. However, its role in lean PCOS is not well studied. This review summarises the potential roles of ExomiR as novel biomarkers that bridge reproductive and non-reproductive facets of PCOS, indicating new paths for precision medicine and tailored treatment interventions in both lean and obese PCOS phenotypes.

## 1 Introduction

PCOS is the most prevalent endocrine disorder in menstruating women and is associated with infertility, metabolic syndrome, obesity, type 2 diabetes, cardiovascular disease, and other long-term consequences (Singh et al., 2023). Recent epidemiological research estimate the worldwide incidence of PCOS to be 5%–20% of women (Bozdag et al., 2016), although the prevalence varies widely depending on the diagnostic criteria, ethnicity, geographic region, and study population. South Asian women (Wolf et al., 2018) seem to bear a disproportionate burden of disease, with prevalence estimates from India (Gupta et al., 2018), Pakistan (Azhar et al., 2020; Rizwan Khan et al., 2024; Zulfiqar et al., 2022), Bangladesh (Kamrul-Hasan et al., 2023) and the Indian subcontinent living in the United Kingdom (Rodin et al., 1998) ranging from about 8% to over 20%, often with more severe metabolic and hyperandrogenic features than in other populations. Despite its high frequency and significant health burden, approximately 70% of afflicted women globally remain misdiagnosed (March et al., 2010).

Beyond reproductive and metabolic sequelae, PCOS is linked with significant losses in standard of living and a higher burden of anxiety, sadness, and psychosocial distress (Lentscher and Decherney, 2021; Alur-Gupta et al., 2024; Vine et al., 2024; Tabassum et al., 2021; Ligocka et al., 2024). The condition also has important long-term healthcare and economic consequences associated with infertility, cardiometabolic disease and mental health concerns (Yadav et al., 2023). In addition, potential shyness among young women to discuss reproductive or sexual health concerns may delay PCOS diagnosis (Ganguly et al., 2025). Delayed diagnosis may be further worsened in many poor and middle-income, even developed, nations by inadequate awareness, sociocultural barriers, limited access to specialist care, and expensive out-of-pocket healthcare expenditures (Vasudevan et al., 2025; Riestenberg et al., 2022; Selvaraj et al., 2018; Rahman et al., 2020). These obstacles underline the importance of accessible and reliable biomarkers for earlier diagnosis, risk classification and appropriate management.

PCOS is defined by elevated androgen levels, irregular ovulation, and the presence of many cysts in the ovaries resulting from complex combinations of genetic, epigenetic, neuroendocrine, metabolic and environmental variables (Repaci et al., 2011). Apart from reproductive problems, PCOS is rapidly becoming recognised as a multisystem condition linked with insulin resistance (Ding et al., 2018), persistent low-grade inflammation, dyslipidaemia, non-alcoholic fatty liver disease and elevated cardiometabolic risk (Owens et al., 2022). Moreover, there is a significant variation between lean and obese PCOS, suggesting the involvement of different pathogenic processes in illness presentation, development and long-term consequences (Moran et al., 2012).

There are numerous criterion sets available for diagnosing PCOS (Figure 1). The most widely used are the Rotterdam criteria (2003), which require at least two of the following three conditions: clinical or biochemical hyperandrogenism, oligo-anovulation, and polycystic ovarian morphology on ultrasound (Christ and Cedars, 2023). Other, less commonly used criteria are the National Institutes of Health (NIH, 1990) (March et al., 2010), necessitates the existence of hyperandrogenaemia and/or hyperandrogenism and oligo/amenorrhea, and the Androgen Excess PCOS Society (2006) (Azziz et al., 2009), which necessitates the presence of hyperandrogenemia and/or hyperandrogenism, and either oligo/amenorrhea or polycystic ovarian morphology.

**FIGURE 1:**
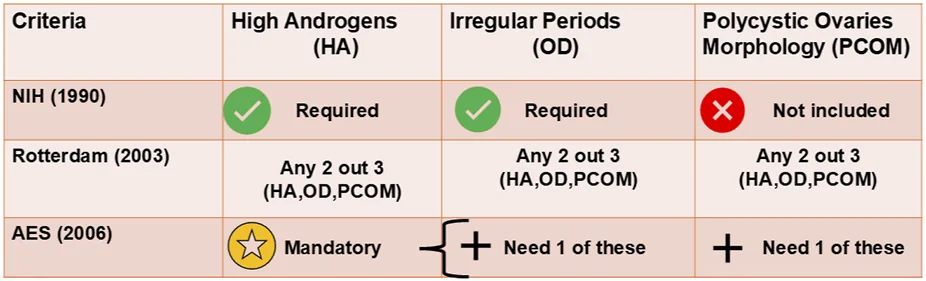
Comparison of NIH (1990), Rotterdam (2003), and AES (2006) diagnostic criteria for PCOS showing the required combinations of hyperandrogenism (HA), ovulatory dysfunction (OD), and polycystic ovarian morphology (PCOM).

The Rotterdam diagnostic criteria demonstrate four distinct phenotypes (A, B, C, D) that show significant variations in Body Mass Index (BMI) and metabolic risk (Shroff et al., 2007). Phenotypes A and B are more severe and have higher BMIs, more insulin resistance, and worse metabolic complications (Sachdeva et al., 2019). Phenotypes C and D are considered milder and have a lower mean BMI and better metabolic characteristics compared to phenotypes A and B (Sachdeva et al., 2019). Lean PCOS affects reproduction and endocrine disorders, whereas obese PCOS is linked with insulin resistance, adipose tissue distribution, chronic inflammation, and increased cardiometabolic risk (Barrea et al., 2021). Due to this heterogeneity, there is an urgent need for molecular markers that link metabolic status with reproductive dysfunction.

Biomarkers are measured biological characteristics that indicate normal or pathological processes or therapeutic response. In PCOS, biomarkers play a critical role in diagnosis, stratification, progression prediction, progression regression, and treatment outcomes (Huffman et al., 2023). Several traditional biomarkers, including luteinizing hormone (LH), follicle-stimulating hormone (FSH), testosterone, and anti-mullerian hormone (AMH), are used to identify the underlying causes of PCOS (Singh et al., 2025a). However, such biomarkers have limitations, such as they become significantly altered only once hormonal dysregulation is established. So, it reflects later stages of PCOS rather than capturing early gene regulation, inflammatory signalling, or inter-organ communication that occur before clinical symptoms develop. Also, those markers have menstrual cycle variability, age dependency, and obesity bias. Moreover, they fail to distinguish different PCOS phenotypes (Saadia, 2020).

Recent investigations have shown that circulating Exosomal miRNA (ExomiR) represents a promising class of minimally invasive biomarkers with improved stability and systemic relevance (Gulati et al., 2024). Exosomes are small extracellular vesicles enclosed in a lipid bilayer, originating from the endosomal pathway (Xu et al., 2022). Early endosomes mature into multivesicular bodies (MVBs) in which proteins, lipids, and nucleic acids, including miRNAs, are selectively integrated (Krylova and Feng, 2023; Mitra et al., 2023). MVBs fuse with the plasma membrane and release exosomes into physiological fluid, including blood, serum, urine, and follicular fluid, thus enabling intercellular communication (Jafari et al., 2023). miRNAs encapsulated in the exosomal lipid bilayer are protected from enzymatic degradation (Xu et al., 2022) and so can stably circulate and be reliably detected. ExomiRs can mediate long-distance inter-organ communication, which suggests that they may represent a common molecular link connecting psychological stress, immune activation, metabolic dysfunction, and cardiovascular disease (Rajan et al., 2024). ExomiRs are important regulators of gene expression and offer biologically significant insights into tissue-specific and systemic disease processes, thereby making them excellent candidates for biomarker development in PCOS (Voros et al., 2025).

In PCOS, ExomiRs could originate from several disease-relevant organs such as the ovary, adipose tissue, liver and immune cells. Thus, ExomiRs may provide a molecular basis to comprehend the physiologic heterogeneity reported among the PCOS manifestations.

ExomiRs are promising biomarkers for PCOS because of their stability, accessibility and ability to capture tissue-specific and systemic changes. The combination of ExomiR profiling with traditional clinical, hormonal and metabolic markers may improve early diagnosis, phenotypic categorisation, risk assessment of multimorbidity and precision-based therapy (Bhome et al., 2018). This review critically appraises the present evidence for ExomiRs as biomarkers in PCOS, focusing on mechanistic relevance and translational potential in lean and obese women (Voros et al., 2025).

## 2 Biomarkers of PCOS

### 2.1 Traditional biomarkers of PCOS

Multiple biomarkers have contributed to understanding the pathophysiology of PCOS, reflecting its reproductive, endocrine, and metabolic disturbances (Singh et al., 2025b). LH, FSH, the LH/FSH ratio, testosterone, dehydroepiandrosterone sulphate (DHEAS), sex hormone-binding globulin (SHBG), and AMHare used to diagnose PCOS (Jiang L. et al., 2023). These biomarkers assess ovarian dysfunction and androgen excess and are routinely used in practice (Jiang L. et al., 2023). Metabolic markers (glucose, insulin, HOMA-IR, OGTT, lipid profile) are useful, particularly in obese PCOS, but may be less sensitive in lean phenotypes (Prosperi and Chiarelli, 2025). In PCOS, inflammatory markers such as C-reactive protein (CRP), interleukins (IL-6, IL-18, TNF-α) and oxidative stress are elevated (Repaci et al., 2011; González, 2012). Some miRNAs, as well as ExomiRs such as miR-21 (Naji et al., 2017), ExomiR-93-3p (Liao et al., 2025), ExomiR-146a-5p (Jiang et al., 2021), ExomiR-155-5p (Cao et al., 2022), miR-222 (Qasemi and Amidi, 2020; Chelegahi et al., 2024) are associated with ovarian function, androgen production, insulin sensitivity, and inflammation, and differ between lean and obese PCOS, suggesting their role as biomarkers of metabolic heterogeneity (Murri et al., 2018). Specifically, ExomiRs regulate important pathways like folliculogenesis (Cao et al., 2022), Steroidogenesis (Azhar et al., 2024), insulin signalling (Jiang et al., 2021) adipogenesis (Hu et al., 2023), inflammation (Yuan et al., 2026) and apoptosis, making them promising biomarkers in PCOS.

### 2.2 Limitations of classical biomarkers

Classical PCOS biomarkers such as LH, FSH, and circulating androgens have limitations, as their levels vary with age, menstrual cycle phase, and assay methods, leading to inconsistent diagnosis and overlap with healthy individuals (Ezeh et al., 2013). For example, AMH, which measures follicle number, has no universally accepted values that can act as a novel biomarker in PCOS. Fasting insulin, glucose, and HOMA-IR, which reflect systemic insulin resistance and cardiometabolic status, but are mostly specific in obese PCOS and less sensitive in lean PCOS (Prosperi and Chiarelli, 2025). In addition, CRP and interleukins are non-specific and influenced by adiposity, resulting in reduced sensitivity in lean PCOS.

## 3 Exosomal miRNAs as novel biomarkers in polycystic ovary syndrome

Exosomes are small extracellular vesicles (30–150 nm) that are released by practically all cell types and discharged into biological fluids, including blood, urine, saliva and follicular fluid (Simons and Raposo, 2009). They transport a plethora of molecular cargo, including proteins, lipids, messenger RNAs and miRNAs to promote communication between distant organs and tissues (Ramachandran and Palanisamy, 2012). Among these types of cargo, ExomiRs have attracted much attention as biomarkers, since they are protected from enzymatic destruction by the exosomal lipid bilayer, and are highly stable under physiological and storage circumstances (Gilad et al., 2008). This stability and their availability through minimally invasive sampling have created a lot of interest in their use as biomarkers for complicated multifactorial disorders such as PCOS.

In contrast to conventional hormonal biomarkers that usually indicate downstream effects of disease, ExomiRs may reveal upstream molecular abnormalities in the earliest stages of disease development (Jafari et al., 2023). Circulating blood ExomiRs offer a unique opportunity to monitor disease activity across many organ systems simultaneously, as PCOS is increasingly recognised as a multisystem condition combining reproductive, metabolic, inflammatory, and neuroendocrine abnormalities (Teede et al., 2026). As exosomes are released from metabolically active tissues, including the ovary, adipose tissue, liver, immune cells, vascular endothelium, and central neuroendocrine tissues, their miRNA cargo may provide an integrated molecular snapshot of the biological processes driving disease heterogeneity and progression (Li et al., 2022).

### 3.1 Exosomal miRNAs reflect the multisystem nature of PCOS

PCOS is characterised by significant phenotypic variation, ranging from exclusively reproductive problems in lean women to severe metabolic dysfunction in obese subjects (Carmina and Lobo, 2022). Conventional biomarkers like as testosterone, LH/FSH ratio, insulin, AMH and inflammatory mediators help provide clinical information but often fail to include the intricate interplay of endocrine, metabolic and immunological pathways (Subashini et al., 2025). Emerging studies suggest that ExomiRs may circumvent some of these constraints by mirroring molecular changes happening in numerous tissues involved in PCOS pathophysiology (Chelegahi et al., 2024). Dysregulated ExomiRs have been linked to crucial biological processes such as ovarian steroidogenesis, follicular development, insulin signalling, adipocyte function, lipid metabolism, oxidative stress, inflammation, and vascular dysfunction (Jiang et al., 2021). Thus, circulating ExomiR profiles may be useful as integrative biomarkers linking reproductive and metabolic aspects of PCOS. Several studies have reported altered expressions of ExomiRs linked with insulin resistance and metabolic dysfunction. For instance, ExomiR-20b-5p is downregulated in PCOS and is predicted to regulate MAPK signalling, endocytosis, and circadian rhythm pathways, suggesting its potential as a biomarker of metabolic dysregulation (Jiang et al., 2021), upregulation of follicular fluid ExomiR-143-3p impairs granulosa cell glycolysis and promotes apoptosis, contributing to follicular dysfunction in PCOS (Cao et al., 2022). These molecular changes are especially relevant in obese PCOS, where insulin resistance is a major contributor to disease severity and long-term cardiometabolic sequelae (Halder et al., 2025; Rojas et al., 2014). Upregulation of adipocyte-derived ExomiR-30c-5p might promote ovarian angiogenesis and PCOS progression via the SOCS3/STAT3/VEGFA signalling pathway (Hu et al., 2023). Within the ovarian microenvironment, upregulated ExomiR-93-3p (Liao et al., 2025) contribute to PCOS-associated alterations in oocyte development and follicular function through modulation of ovarian signalling networks, including PI3K/Akt, MAPK and TGF-β pathways. Their altered expression, particularly in hyperandrogenic PCOS, highlights their potential as diagnostic and mechanistic biomarkers.

Aberrant expression of these ExomiR may lead to follicular arrest and hyperandrogenism, two hallmarks of PCOS. Thus, ovarian-derived ExomiRs may provide mechanistic insight into reproductive failure and may also serve as potential indicators of disease activity.

### 3.2 Clinical significance of exosomal miRNAs in lean and obese PCOS

One of the biggest hurdles in managing PCOS is to identify clinically useful biomarkers that can distinguish between lean and obese phenotypes. Several women with normal body weight have reproductive dysfunction, insulin resistance and elevated cardiometabolic risk despite the absence of overt obesity (Carmina and Lobo, 2022). But obesity worsens several features of PCOS. Therefore, traditional clinical criteria alone may underestimate the severity of disease in some patient populations.

ExomiRs are an exciting solution as their expression profiles seem to reflect phenotype-specific disease processes. In lean PCOS, it is believed that reproductive and neuroendocrine problems predominate (Carmina and Lobo, 2022). Ovarian granulosa and theca cell-secreted exosomes include miRNAs that participate in steroidogenesis, androgen biosynthesis, follicular development, and insulin signalling (Zhang et al., 2023). Upregulation of miR-21 (Naji et al., 2017) and ExomiR-93-3p (Liao et al., 2025), downregulation of miR-145 (Cai et al., 2017) contributes to impaired folliculogenesis in PCOS by regulating the IRS1/PI3K–AKT signalling pathway, highlighting its potential as an ovarian biomarker, whereas ExomiR-379-5p is decreased in PCOS follicular fluid exosomes and regulates granulosa cell proliferation through the PDK1/PI3K-AKT pathway (Salehi et al., 2023). Downregulation of ExomiR-320 contributes to insulin resistance and ovarian dysfunction by targeting the PI3K-AKT signalling pathway, highlighting its potential as a mechanistic biomarker in hyperinsulinemic PCOS, suggesting its potential as a biomarker and therapeutic target for ovarian dysfunction (Chelegahi et al., 2024) (Table 1).

**TABLE 1:** MicroRNA/Exosomal microRNAs in polycystic ovary syndrome**:** Summary of microRNA(miR) or exosomal microRNAs (ExomiRs) associated with PCOS, including their expression pattern, biological source, major target/pathway, associated clinical features, lean/obese association, and biomarker potential. Expression is shown relative to healthy controls (↑ upregulated; ↓ downregulated). Abbreviations: ExomiR, exosomal microRNA; EV, extracellular vesicle; FF, follicular fluid; GC, granulosa cell.

microRNA(miRNA)/ Exosomal miRNA (ExomiR)	Expression in PCOS	Source (plasma/serum/ follicular fluid)	Major target/pathway	Associated clinical feature	Lean/obese association	Bio-marker potential	References
ExomiR-379-5p	↓Decreased in follicular fluid exosomes; androgen promotes its exosomal release from preantral granulosa cells, reducing intracellular miR-379-5p	Follicular fluid exosomes	PDK1→ PI3K/AKT signalling, regulation of granulosa cell proliferation and follicular development	Hyperandrogenism, impaired granulosa cell proliferation, follicular arrest, ovarian dysfunction in PCOS	Not stratified into lean or obese PCOS	Promising diagnostic and therapeutic ExomiR for androgen-mediated ovarian dysfunction; requires clinical validation	Salehi et al. (2023)
ExomiR-143-3p	↑ Upregulated	Follicular fluid-derived exosomes	Glycolysis regulation; granulosa cell (GC) metabolism; promotes GC apoptosis	Follicular dysplasia, impaired folliculogenesis, PCOS pathogenesis	Not evaluated (no lean vs. obese subgroup analysis)	Promising diagnostic and therapeutic biomarker	Cao et al. (2022)
ExomiR-155-5p	↓ Downregulated	Follicular fluid-derived exosomes	Glycolysis regulation; granulosa cell metabolism	Reduced glycolytic activity and altered GC function	Not reported	Potential biomarker of follicular dysfunction	Cao et al. (2022)
ExomiR-424-5p	Dysregulated in PCOS follicular fluid; lower ExomiR-424-5p was associated with higher pro-inflammatory cytokines	Follicular fluid-derived exosomes	FGF2/FGFR1 signaling; miR-424-5p directly targets FGF2	Ovarian inflammation, granulosa-cell dysfunction and ovulatory impairment	Not evaluated	Potential biomarker/therapeutic candidate for ovarian inflammation and ovulatory dysfunction in PCOS	Yuan et al. (2026)
	↓ Downregulated in follicular-fluid exosomes from PCOS patients	Follicular fluid extracellular vesicles (FFEVs/exosomes)	CDCA4 → Rb/E2F1 pathway; miR-424-5p targets CDCA4	Granulosa-cell senescence, reduced proliferation and impaired follicular development/ovulatory dysfunction	Not established in Duval et al. (2024)	Potential biomarker and therapeutic candidate for PCOS-related follicular dysfunction	Duval et al. (2024)
ExomiR-20b-5p	↓ Downregulated	Plasma exosomes	MAPK signaling, endocytosis, circadian rhythm (predicted)	Associated with plasma steroid hormones; potential role in insulin resistance	Not evaluated	Moderate–High	Jiang et al. (2021)
ExomiR-30c-5p	↑ Upregulated	Adipocyte-derived exosomes (functional transfer to ovarian tissue; increased ovarian miR-30c-5p in PCOS mice)	SOCS3 → STAT3 → VEGFA signaling pathway; miR-30c-5p directly targets SOCS3, promoting STAT3 activation and VEGFA expression	Enhanced ovarian angiogenesis, endothelial cell proliferation, migration, tube formation, insulin resistance, endocrine abnormalities, PCOS progression	Not evaluated	Promising therapeutic and mechanistic biomarker; potential target for anti-angiogenic therapy	Hu et al. (2023)
miR-21	↑ Increased in PCOS (granulosa cells and follicular fluid)	Granulosa cells and follicular fluid (ovarian follicular microenvironment)	PTEN/PI3K-AKT signalling pathway; apoptosis regulation; cell survival and proliferation pathways in granulosa cells	Follicular dysfunction, altered granulosa cell proliferation/apoptosis balance, impaired folliculogenesis, and association with PCOS phenotypes (including hyperandrogenic and ovulatory dysfunction features)	Not stratified by lean vs. obese PCOS; expression differences reported across PCOS phenotypes rather than	Potential diagnostic and mechanistic biomarker of granulosa cell dysfunction and follicular microenvironment dysregulation in PCOS	Naji et al. (2017)
ExomiR-93-3p	↑ Increased in follicular fluid extracellular vesicles in PCOS	Follicular fluid extracellular vesicles (FF-EVs) derived from ovarian follicular microenvironment in women with PCOS	PI3K/AKT signaling dysregulation; oocyte maturation and meiotic progression pathways; granulosa–oocyte communication impairment	Oocyte developmental arrest, impaired meiotic progression, reduced oocyte quality and fertility dysfunction in PCOS	Not stratified by lean vs. obese PCOS in this study; elevation observed across PCOS cohort compared	Potential diagnostic and mechanistic biomarker for PCOS-related oocyte dysfunction; candidate marker of follicular microenvironment disturbance	Liao et al. (2025)
miR-145	↓ Downregulated in granulosa cells from women with PCOS	Granulosa cells/follicular fluid	IRS1, PI3K/AKT signaling	Impaired folliculogenesis	No clear lean/obese distinction but irrespective of obesity	Candidate ovarian biomarker	Cai et al. (2017)
ExomiR-146a-5p	↑ Upregulated	Plasma exosomes	MAPK signaling, endocytosis, axon guidance and circadian-rhythm-related pathways; target analysis also implicates BRCA1/BRCA2-related signaling	Associated with PCOS phenotype, including menstrual-cycle characteristics, antral follicle count and hormone levels	did not establish a lean-vs-obese PCOS association	Potential circulating diagnostic biomarker; five-miRNA panel showed AUC = 0.781 for distinguishing PCOS from controls	Jiang et al. (2021)
miR-222	↓ Decreased in HA-PCOS and NA-PCOS compared with normal controls; HA-PCOS showed lower levels than NA-PCOS	Follicular fluid	Estrogen signaling/steroidogenesis; ESR1 and PTEN	Steroidogenesis and endocrine/reproductive dysfunction	Reported difference between HA-PCOS and NA-PCOS; however, HA/NA refers to hyperandrogenic/non-hyperandrogenic PCOS, not obese/lean	Potential follicular-fluid biomarker for PCOS phenotype/reproductive dysfunction, but requires validation	Qasemi and Amidi (2020)
	↑ Increased in serum of women with PCOS	Serum; non-exosomal/circulating	Endocrine and metabolic pathways; regulation of estradiol; associated with insulin signaling	Hyperandrogenism, insulin resistance and altered estradiol regulation	Not specifically evaluated as lean vs. obese PCOS	Potential circulating biomarker for PCOS and associated metabolic/endocrine abnormalities	Chelegahi et al. (2024)
ExomiR-320	↓ Decreased in PCOS	Follicular fluid–derived exosomes (granulosa cell–oocyte microenvironment)	Insulin signaling pathway (e.g., IGF-1/PI3K-AKT axis regulation), glucose metabolism, and granulosa cell proliferation/apoptosis balance	Impaired folliculogenesis, granulosa cell dysfunction, reduced oocyte quality, and metabolic dysregulation within ovarian follicular environment in PCOS	Not stratified by lean vs. obese PCOS in the review; reported alteration considered PCOS-related rather than BMI-specific	Potential diagnostic and mechanistic biomarker of follicular dysfunction and insulin-related dysregulation in PCOS ovarian microenvironment	Chelegahi et al. (2024)
ExomiR-126-3p	Differentially expressed in PCOS plasma exosomes	Plasma-derived exosomes	PDGFRβ → PI3K/AKT signaling; predicted/validated functional effects on granulosa cells	Granulosa-cell proliferation, hormone synthesis and reproductive dysfunction; expression associated with menstrual cycle, antral follicle count and hormone levels	Not evaluated	Promising candidate biomarker for PCOS; an exosomal miRNA panel including miR-126-3p showed diagnostic potential	Jiang et al. (2021); Jiang et al. (2023b)

Several circulating and ExomiRs have been implicated in PCOS and its associated metabolic abnormalities. Plasma ExomiR-126-3p was reported to be differentially expressed in women with PCOS and correlated with reproductive and hormonal features (Jiang et al., 2021; Jiang X. et al., 2023). Together, these findings suggest that circulating and ExomiR profiles may constitute promising candidates for identification of metabolic dysfunction and stratification of individuals with PCOS that may be at increased metabolic risk.

Obese PCOS, in contrast, is characterised by visceral obesity, chronic low-grade inflammation, dyslipidemia and insulin resistance. ExomiR-146a-5p is dysregulated in PCOS and is associated with inflammatory and metabolic pathways including NF-κB signalling, endothelial dysfunction, lipid metabolism and glucose homeostasis (Jiang et al., 2021). Conversely, ExomiR-424-5p is downregulated in follicular-fluid exosomes of women with PCOS, which may control ovarian inflammation via the FGF2/FGFR1 axis, thus demonstrating its significance as a diagnostic and therapeutic biomarker. Importantly, the study did not stratify participants by lean versus obese PCOS (Yuan et al., 2026; Duval et al., 2024) (Table 1).

Significantly, patient classification could be molecularly based on phenotype-specific ExomiR signals. ExomiR profiling may assist in classifying individuals not by PCOS as a medical entity, but by their major biological anomalies like ovarian dysfunction, insulin resistance, inflammation or neuroendocrine dysregulation. This strategy is well in line with growing ideas in precision medicine to target the development of therapies based on disease mechanisms in the individual.

### 3.3 Diagnostic, prognostic and translational potential of exosomal miRNAs

ExomiRs have clinical significance beyond detection of illness. As changes in ExomiR levels have been seen before the appearance of clear symptoms such as menstrual irregularity, obesity, hyperandrogenism and poor glucose tolerance, they can be used as early markers of disease predisposition and progression (Esfandyari et al., 2021). This is especially pertinent in teenagers and young women when standard diagnostic criteria might be difficult to interpret, and late diagnosis is frequent (Tay et al., 2020).

ExomiRs may also have a prognostic usefulness beyond the diagnosis. Specific ExomiR signatures may have the potential to identify women at elevated risk for development of insulin resistance (Vukelić et al., 2026), Gestational Diabetes Mellitus (GDM) (Mitra et al., 2024), type 2 diabetes mellitus, cardiovascular disease (Xu et al., 2023), non-alcoholic fatty liver disease (Yang et al., 2025), infertility (Voros et al., 2025) and endometrial pathology (Sykaras et al., 2022). Monitoring of circulating ExomiRs longitudinally may give insight into illness progression and therapy response (Théry, 2011). Another key feature of ExomiRs is the possibility of using them for treatment monitoring. Changes in ExomiR profiles can signal biological responses to lifestyle changes, insulin-sensitising medications, anti-androgen therapy or new targeted medicines. Thus, ExomiRs can serve as dynamic biomarkers for real-time assessment of treatment efficacy.

While conventional hormonal, metabolic, and inflammatory biomarkers provide partial insights into PCOS pathology, they lack specificity and sensitivity for early and phenotype-specific diagnosis. ExomiR overcome these limitations through enhanced stability, biological relevance, and non-invasive accessibility. The Earth Mover’s Distance (EMD) model (Shrivastav et al., 2022) can be applied in PCOS, where ExomiRs act as indicators along a continuum of ovum normality to abnormality, aligning ExomiR expression patterns with increasing PCOS severity (Figure 2). This framework supports the notion that ExomiRs not only reflect underlying metabolic and reproductive disturbances but may also serve as early screening tools and triaging markers for stratifying PCOS severity. This approach supports precision-based stratification, early triaging, and community-level screening of women with varying PCOS phenotypes.

**FIGURE 2:**
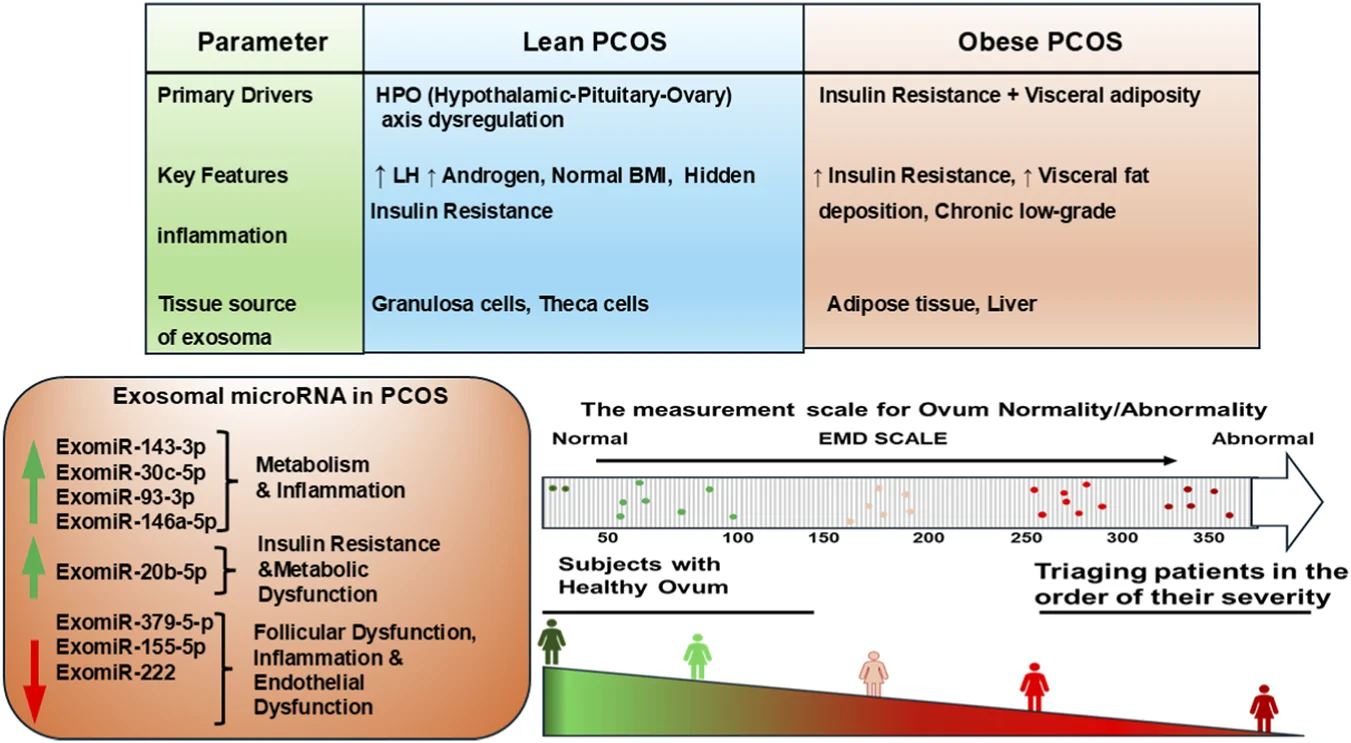
Exosomal microRNA acts as an early screening and triaging of PCOS severity: Circulating ExomiR signatures differentiate Lean and Obese PCOS phenotypes and enable early screening and severity-based triaging through a proposed molecular dysregulation scale.

ExomiRs are a promising class of non-invasive biomarkers that bridge reproductive and metabolic features of PCOS. They represent tissue-specific molecular changes and inter-organ communication networks, providing a physiologically informative platform for studying disease heterogeneity and enhancing precision treatment methods in both lean and obese PCOS (Voros et al., 2025).

## 4 Beyond the ovary: multisystem impact of PCOS

PCOS is increasingly recognised as a systemic disorder that extends far beyond ovarian dysfunction and reproductive abnormalities. The diagnostic features of PCOS include hyperandrogenism, ovulatory dysfunction and polycystic ovarian morphology. Accumulating evidence indicates that the syndrome involves extensive interactions among neuroendocrine, metabolic, immune, hepatic, and cardiovascular systems (Sadeghi et al., 2022). Importantly, the relative contribution of these pathways differs between lean and obese PCOS phenotypes, resulting in distinct clinical presentations and long-term health risks (Carmina and Lobo, 2022).

### 4.1 Neuroendocrine and metabolic heterogeneity in lean and obese PCOS

Mechanisms underlying PCOS are very different between phenotypes. Lean PCOS seems more related to neuroendocrine dysfunction and intrinsic ovarian malfunction due to the elevation of GnRH pulse frequency from the hypothalamus, which causes more LH than FSH to be released, which overstimulates theca cells and leads to excess androgen production (Pratama et al., 2024). Obese PCOS is defined mainly by metabolic disturbance of visceral obesity and insulin resistance.

In lean PCOS, reproductive dysfunction is often shown in normal body weight without overt metabolic illness. Genetic predisposition, aberrant steroidogenesis, inappropriate gonadotropin secretion and problems in post-receptor insulin signalling have all been implicated in the pathophysiology of illness (Figure 3). While insulin binding to its membrane receptor is preserved, subsequent intracellular signalling cascades, particularly those related to glucose transporter (GLUT4) translocation, are compromised. This post-receptor malfunction restricts cellular glucose absorption despite sufficient receptor activation. In reaction, pancreatic β-cells augment insulin production, leading to hyperinsulinemia. Chronic hyperinsulinemia has considerable endocrine implications. Elevated insulin decreases hepatic synthesis of sex hormone-binding globulin (SHBG), thereby increasing circulating free testosterone levels (Toosy et al., 2018). The elevated level of androgens leads to clinical hyperandrogenic symptoms, such as acne, alopecia, and hirsutism. Notably, lean PCOS often demonstrates a strong genetic and epigenetic predisposition, indicating that inherited alterations in insulin signalling, steroidogenesis, and inflammatory pathways underlie disease susceptibility. On the other hand, in obese PCOS, adipose tissue acts as an endocrine organ. In this case, it displays a specific type of insulin resistance, where insulin is unable to effectively prompt GLUT-4-mediated glucose absorption. It causes severe insulin resistance, hyperinsulinemia and dysfunctional adipose tissue, which aggravate reproductive and endocrine problems (Ezeh et al., 2019; Ezeh et al., 2020).

**FIGURE 3:**
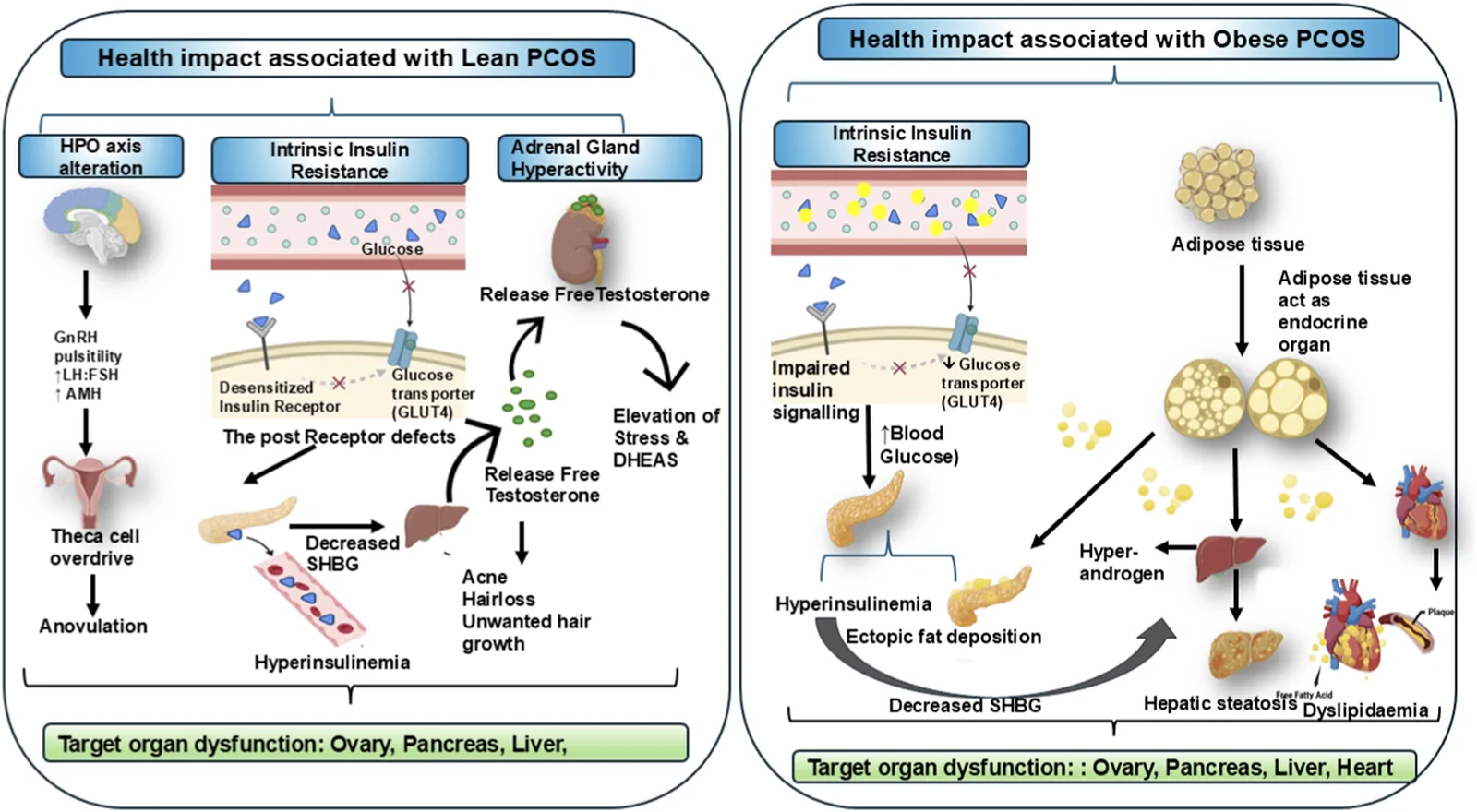
Health impact associated with lean and obese PCOS: Lean PCOS is mainly associated with HPO axis dysregulation, intrinsic insulin resistance, and adrenal hyperactivity, leading to hyperandrogenism and reproductive dysfunction. Obese PCOS is characterized by adipose tissue–driven insulin resistance, hyperinsulinemia, and metabolic complications, including hepatic steatosis and dyslipidemia, contributing to multi-organ dysfunction.

### 4.2 Immune dysregulation and chronic inflammation

Chronic low-grade inflammation is becoming acknowledged as a factor in PCOS pathogenesis. Expansion of adipose tissue in obese PCOS enhances macrophage infiltration and release of inflammatory mediators at high levels, including TNF-α, IL-6, MCP-1 and leptin. These cytokines disrupt insulin signalling and promote oxidative stress, which leads to endothelial dysfunction and cardiometabolic consequences (Dimitriadis et al., 2016).

Although lean PCOS is often associated with lower levels of systemic inflammation, oxidative stress, altered immune-cell activation, and low-grade inflammatory signals have been reported in various investigations, irrespective of obesity. These data show that immunological dysregulation may be a common hallmark of PCOS, with the severity and underlying mechanisms differing between phenotypes (Keskin Kurt et al., 2014).

### 4.3 Hepatic and cardiovascular consequences

PCOS is associated with systemic problems such as hepatic and cardiovascular abnormalities beyond the reproductive system (Ollila et al., 2023). The major factors that may contribute to the elevated risk of non-alcoholic fatty liver disease (NAFLD), dyslipidemia, hypertension, endothelial dysfunction, and cardiovascular disease in obese women with PCOS include insulin resistance and chronic inflammation (Keskin Kurt et al., 2014).

Importantly, lean PCOS cannot be regarded as metabolically benign. There is increasing evidence that normal-weight women with PCOS may have endothelial dysfunction, decreased vascular reactivity, and unfavorable lipid profiles compared to healthy controls (Toosy et al., 2018). These observations point to the need for long-term cardiometabolic surveillance regardless of body mass index.

### 4.4 Exosomal microRNAs as mediators of inter-organ communication

The molecular processes underlying the connection between reproductive failure and systemic metabolic and cardiovascular problems are still partially understood. Recent research indicates that ExomiRs may be crucial players as mediators of communication between the ovary, adipose tissue, liver, immune system and vascular endothelium (Isaac et al., 2021).

ExomiRs regulate several pathways involved in PCOS steroidogenesis, insulin signalling, glucose metabolism, adipogenesis, inflammation, oxidative stress and endothelial function. Ovarian ExomiRs (Salehi et al., 2023) may largely represent anomalies in follicular growth and androgen synthesis, while adipose tissue (Mei et al., 2022) and liver ExomiRs (Noh et al., 2025) appear to be more closely linked to insulin resistance, lipid metabolism and inflammatory responses. The function of ExomiRs to circulate between organs and modulate gene expression in distant tissues may offer a mechanistic link between reproductive failure and systemic multimorbidity.

Importantly, phenotype-specific ExomiRs signatures may elucidate the distinctions between lean and obese PCOS. ExomiRs involved with steroidogenesis and neuroendocrine control might be more prevalent in lean PCOS (Zheng et al., 2025), whereas ExomiRs related to metabolic and inflammatory pathways might be dominant in obese PCOS (Voros et al., 2025). Thus, ExomiR profiling may provide insight into illness heterogeneity, as well as a biomarker technique for phenotypic categorisation and risk prediction.

Together, these findings are consistent with the hypothesis that PCOS is a multisystem illness where reproductive, metabolic, immunological, hepatic and cardiovascular disorders are linked by complex inter-organ communication networks (Venkatraman et al., 2025). ExomiRs might offer a biological basis for the association between these different symptoms and could also contribute to the phenotypic variability reported between lean and obese PCOS. Their ability to represent tissue-specific dysfunction across organ systems underscores their potential utility as biomarkers for disease classification and multimorbidity risk assessment.

## 5 Neuroendocrine dysregulation and exosomal microRNAs in PCOS

Neuroendocrine dysfunction is a central component of PCOS pathophysiology (Burt Solorzano et al., 2012) and arises from complex interactions among the hypothalamic-pituitary-ovarian (HPO), hypothalamic-pituitary-thyroid (HPT) and hypothalamic-pituitary-adrenal (HPA) axes (Kadhim and Kuenzel, 2022; Mikhael et al., 2019). These interrelated systems regulate reproductive function, metabolic balance, stress responses and inflammatory pathways and are likely to explain the substantial variation reported between PCOS symptoms. Recent data suggest that ExomiRs may be involved in crosstalk between the endocrine axis and peripheral target tissues, hence suggesting a biological link between neuroendocrine failure and systemic features of PCOS (Voros et al., 2025).

### 5.1 HPO axis dysfunction: central driver of reproductive abnormalities

Abnormal gonadotropin-releasing hormone (GnRH) pulsatility is a characteristic of PCOS (McCartney and Campbell, 2020). Increased GnRH pulse frequency selectively promotes the secretion of LH and reduces the secretion of FSH and thereby increases the LH/FSH ratio. High LH signalling favours ovarian theca-cell androgen synthesis. Relatively low FSH restricts granulosa-cell aromatisation of androgens to estrogens. The hyperandrogenic milieu thus created leads to follicular arrest, anovulation and menstrual abnormalities (Zeng et al., 2022).

Hyperinsulinemia exacerbates HPO-axis dysfunction by boosting ovarian steroidogenesis and reducing hepatic synthesis of sex hormone-binding globulin (SHBG), hence raising free androgen concentrations in the circulation (Liao et al., 2021). These connections produce a positive feedback loop between metabolic disorders and reproductive problems.

Several ExomiRs are involved in granulosa-cell proliferation, follicular growth, and steroidogenesis dysregulated in PCOS. These ExomiR delivered by exosomes might mediate communication within the ovarian milieu and contribute to modified ovarian function. Therefore, ExomiRs from the ovary may be potential indicators for the severity of reproductive failure and ovarian disease.

### 5.2 HPT axis: a potential modifier of metabolic and reproductive dysfunction

Thyroid dysfunction is not a major cause of PCOS, although evidence is mounting that it might affect disease severity. Thyroid hormones affect energy expenditure, lipid metabolism, glucose homeostasis and reproductive physiology. Elevated thyroid-stimulating hormone (TSH) levels are related to poor metabolic profiles and increased insulin resistance in individuals with PCOS (Cai et al., 2019).

There is a bidirectional relationship between thyroid and ovarian function. Ovarian steroid hormones can affect thyroid hormone metabolism and pituitary reactivity, and hypothyroidism can cause menstrual abnormalities by inducing hyperprolactinemia and changes in GnRH output. Thus, thyroid dysfunction might worsen reproductive and metabolic problems that are already evident in PCOS (Palomba et al., 2023).

Studies directly trying ExomiRs to HPT-axis dysfunction in PCOS are still sparse, however some circulating ExomiR have been linked to thyroid hormone signalling, inflammatory pathways and metabolic control. Further studies may clarify whether ExomiR signatures can identify women in whom thyroid dysfunction is a contributor to illness progression and cardiometabolic risk.

### 5.3 HPA axis and stress-related mechanisms

The HPA axis is another neuroendocrine mechanism that could contribute to phenotypic heterogeneity in PCOS. In a subset of women with PCOS, there have been observed increases in adrenal responsiveness to adrenocorticotropic hormone (ACTH), leading to heightened adrenal androgen production, including dehydroepiandrosterone (DHEA) and dehydroepiandrosterone sulphate (DHEAS) (Ring, 2025). It is thought that 20%–30% of affected women have functional adrenal hyperandrogenism, which may be responsible for both hyperandrogenism and ovulatory dysfunction (Torchen et al., 2025).

Altered cortisol dynamics have also been found in PCOS. Chronic psychological stress, metabolic stress and inflammation can affect the activity of HPA-axis, but the results are still inconsistent (Gianotti et al., 2021). Lean PCOS seems more associated with increased neuroendocrine sensitivity and adrenal androgen excess, while obese PCOS may have prolonged activation of stress-related pathways secondary to insulin resistance and adipose tissue inflammation (Carmina et al., 2005).

The interplay between the HPA and HPO axis is particularly relevant, since glucocorticoids and adrenal androgens can alter reproductive hormone production and ovarian function (Gianotti et al., 2021). ExomiRs associated with inflammatory and stress-sensitive pathways may also be important in communication across adrenal, metabolic and reproductive organs, however, direct data are scarce (Figure 4).

**FIGURE 4:**
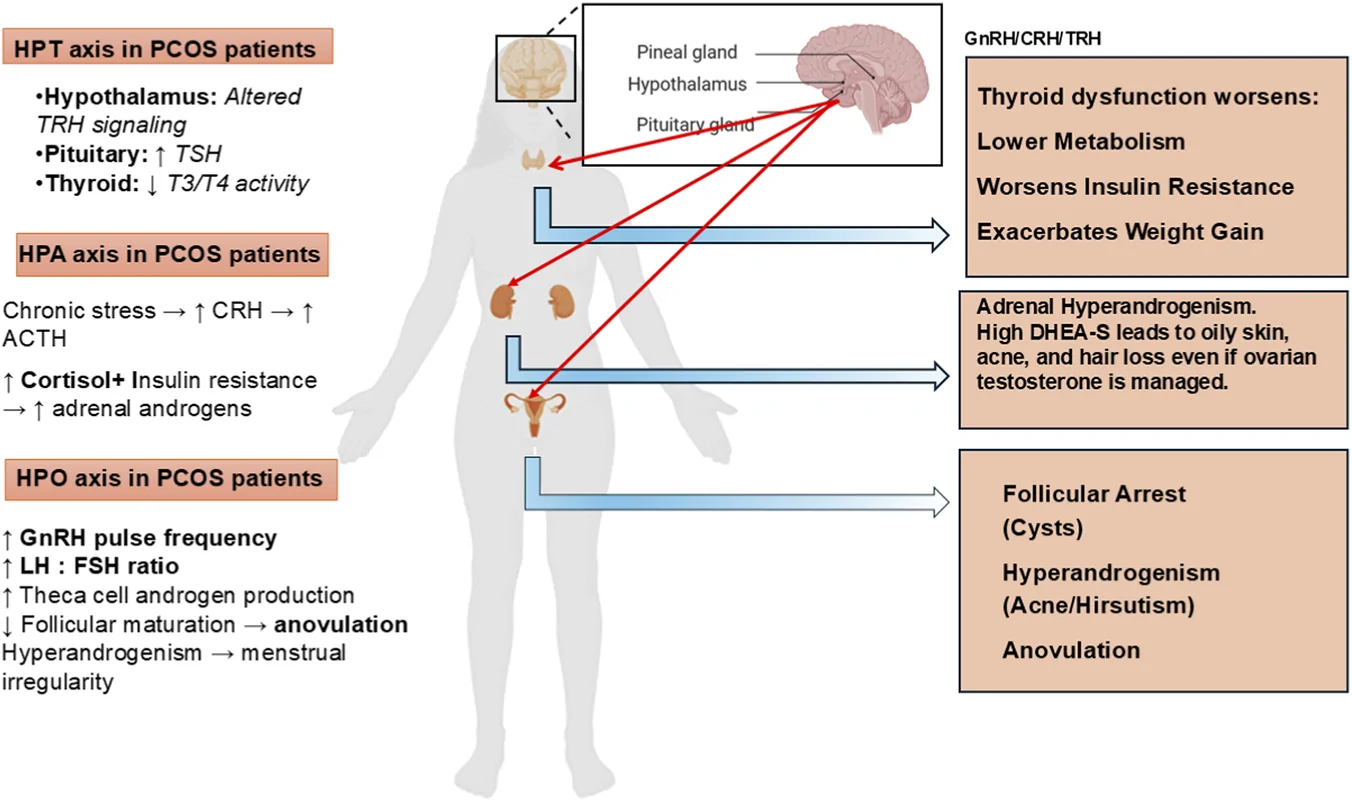
Role of HPT, HPO and HPA axis in PCOS: Dysregulation of the HPT, HPA, and HPO axes in PCOS contributes to thyroid dysfunction, stress-related adrenal hyperandrogenism, insulin resistance, follicular arrest, and anovulation, driving the reproductive and metabolic manifestations of the syndrome.

In summary, the HPO, HPT and HPA axis are involved in the reproductive, metabolic and stress-related features of PCOS. ExomiRs, are promising integrative biomarkers that can better capture disease heterogeneity than individual measures that can reflect molecular abnormalities in these interrelated endocrine systems. Future work combining ExomiR profiling with endocrine and metabolic phenotyping may improve earlier diagnosis, phenotypic stratification, prediction of multimorbidity risk and individualised treatment approaches in women with both lean and obese PCOS.

## 6 Conclusion

Evaluation of circulating ExomiR as a biomarker of multimorbidity risk in lean and obese PCOS will provide new insights into disease stratification and long-term clinical management. Further research on ExomiR profiling with endocrine, metabolic, and inflammatory parameters may facilitate early triaging of PCOS phenotypes with distinct risk factors, even before overt clinical manifestations emerge. Such approaches may help to predict comorbidities such as insulin resistance, type 2 diabetes mellitus, cardiovascular disease, non-alcoholic fatty liver disease, and endometrial pathology in both lean and obese PCOS populations.

Longitudinal and multi-omics investigation will help to validate phenotype-stratified ExomiR that reflects inter-organ communication between the ovary, adipose tissue, liver, immune system, and central neuroendocrine axes. These signatures may serve as stable, non-invasive biomarkers for monitoring disease progression, treatment response, and transition from reproductive dysfunction to systemic multimorbidity across the lifespan of women with PCOS. From a translational perspective, future outcomes include the development of precision medicine strategies in which ExomiRs panels guide personalised interventions based on individual risk profiles. In lean PCOS, such biomarkers may help elucidate neuroendocrine and stress-induced disease mechanisms, whereas in obese PCOS, they may enable the early identification of metabolic and inflammatory complications.

Overall, ExomiRs as biomarkers are expected to shift PCOS management from a symptom-centred approach to a predictive, preventive, and personalised model of care, ultimately reducing multimorbidity burden and improving long-term reproductive and cardiometabolic outcomes in both lean and obese PCOS phenotypes.

## 7 Future direction

ExomiR has become highlighted as a promising molecular intermediary of intercellular communication and modulator of metabolic and inflammatory pathways. In PCOS, increasing evidence suggests that circulating ExomiR expression is different in obese PCOS compared to healthy controls. It reflects metabolic and endocrine dysfunction. Lean PCOS has been comparatively underexplored since most of the research on PCOS has traditionally concentrated on obesity. There is insufficient evidence about circulating ExomiR signatures and their association with cardiometabolic and inflammatory risk in lean PCOS. Integrating ExomiR profiling with clinical parameters, immune signatures, and vascular markers such as Carotid Intima-Media Thickness could lead to the development of multimodal precision-screening strategies for early detection of cardiometabolic risk in lean and obese PCOS patients. Furthermore, understanding the physiological activities of ExomiRs may improve risk stratification and management of PCOS-associated multimorbidity.
